# d-Dimer elevation and adverse outcomes

**DOI:** 10.1007/s11239-014-1101-6

**Published:** 2014-07-09

**Authors:** Rim Halaby, Christopher J. Popma, Ander Cohen, Gerald Chi, Marcelo Rodrigues Zacarkim, Gonzalo Romero, Samuel Z. Goldhaber, Russell Hull, Adrian Hernandez, Robert Mentz, Robert Harrington, Gregory Lip, Frank Peacock, James Welker, Ignacio Martin-Loeches, Yazan Daaboul, Serge Korjian, C. Michael Gibson

**Affiliations:** 1 Cardiovascular Division, Department of Medicine, Beth Israel Deaconess Medical Center, Harvard Medical School, East Campus, RW 459, Boston, MA 02215 USA; 2 King’s College Hospital, London, UK; 3 Cardiovascular Division, Department of Medicine, Brigham and Women’s Hospital, Harvard Medical School, Boston, MA USA; 4 Department of Medicine Calgary, Foothills Hospital, Calgary, Canada; 5 Division of Cardiology, Department of Medicine, Duke University Medical Center, Durham, NC USA; 6 Department of Medicine, Stanford University, Stanford, CA USA; 7 Centre for Cardiovascular Sciences, City Hospital, University of Birmingham, Birmingham, UK; 8 Emergency Department, Baylor College of Medicine, Houston, TX USA; 9 Anne Arundel Medical Center, Anne Arundel Health System Research Institute, University of Maryland School of Medicine, Baltimore, MD USA; 10 Critical Care Centre, Corporació Sanitària I Universitària Parc Taulí Hospital De Sabadell Parc Taulí, Barcelona, Spain

**Keywords:** d-Dimer, Deep vein thrombosis, Mortality, Prognosis, Pulmonary embolism

## Abstract

d-Dimer is a biomarker of fibrin formation and degradation. While a d-dimer within normal limits is used to rule out the *diagnosis* of deep venous thrombosis and pulmonary embolism among patients with a low clinical probability of venous thromboembolism (VTE), the *prognostic* association of an elevated d-dimer with adverse outcomes has received far less emphasis. An elevated d-dimer is independently associated with an increased risk for incident VTE, recurrent VTE, and mortality. An elevated d-dimer is an independent correlate of increased mortality and subsequent VTE across a broad variety of disease states. Therefore, medically ill subjects in whom the d-dimer is elevated constitute a high risk subgroup in which the prospective evaluation of the efficacy and safety of antithrombotic therapy is warranted.

## Introduction

d-Dimer is a biomarker of fibrin formation and degradation [1–3]. It may be elevated not only in patients with acute thrombosis, but also among the elderly as well as in a variety of illnesses [4, 5]. While a d-dimer within normal limits is used to rule out the *diagnosis* of deep venous thrombosis and pulmonary embolism among patients with a low clinical probability of venous thromboembolism [6, 7], the *prognostic* association of an elevated d-dimer with adverse outcomes has received far less emphasis.

## Methods

A search of the Pubmed and Cochrane databases was independently performed by four individuals. The keyword used for the search was d-dimer in combination with other words such as prognosis, mortality, recurrence, incidence, and occurrence. The search was not limited by country or language and it included prospective and retrospective observational studies, clinical trials and systematic reviews. Study selection was initially based on the review of the title and it yielded a total of 87 articles. The articles were categorized as follows (some articles fit more than one category): d-dimer and mortality, d-dimer and occurrence of VTE, and d-dimer and recurrence of VTE. The four individuals reviewed the articles for quality assessment and extracted information about the study population, study design, statistical analysis, outcomes (symptomatic DVT, asymptomatic DVT, PE, VTE and mortality), results and limitations. Based on their methodology, adequacy of their sample size, and statistical analysis, a total of 37 articles were considered of at least average quality and were included in this manuscript.

## d-Dimer and subsequent occurrence of VTE

Among acutely and critically ill patients, an elevated d-dimer is associated with an increased risk of a subsequent occurrence of a first episode of VTE independent of age, sex, race, body mass index, and medical illnesses [8–12]. In fact, among 7581 acutely ill medical patients enrolled in the MAGELLAN study, the incidence of a subsequent VTE was 3.5 times higher in patients with baseline concentrations of d-dimer equal or greater than two times the upper limit of normal compared to those with normal d-dimer (OR 2.29; 95 % CI 1.75–2.98) [12]. In the general population, an elevated d-dimer is associated with an increased risk of a subsequent first episode of VTE among 923 subjects during 8 years of follow-up [13].

In various disease states, d-dimer is associated with the subsequent first occurrence of VTE and adverse clinical outcomes. During ischemic stroke, there is activation of the coagulation and fibrinolytic systems which may be reflected by elevations in d-dimer [14, 15]. In a prospective multi-center study including 1,380 stroke patients followed for 12 months, an elevated d-dimer measured at the time of hospitalization was independently associated with the subsequent first occurrence of VTE (OR 3.45; 95 % CI 2.01–8.52) [15].

As a specific pathologic state, cancer is associated with a higher risk of subsequent first occurrence of VTE, a risk further amplified by hospitalization, surgical intervention, or chemotherapy [16]. d-Dimer is significantly higher among cancer patients who subsequently develop a first episode of VTE compared to those who do not [17, 18]. Among 821 cancer patients with a twofold increase in d-dimer concentration, there was a 1.3 fold increase in the first occurrence of VTE both in univariate (95 % CI 1.1–1.5; *p* < 0.001) and multivariate analyses (HR 1.3; 95 % CI 1.2–1.6; *p* < 0.001) following adjustment for age, sex, radiotherapy, chemotherapy, and surgery [17]. The association of an elevated d-dimer with the first occurrence of VTE has been validated in multiple studies among patients with a variety of oncologic pathologies including colorectal, lung, and gynecological cancer [19–23].

## d-Dimer and recurrence of VTE

While the previous studies evaluated the association of d-dimer with the first occurrence of VTE, recurrent VTE following an initial episode is associated with an elevated d-dimer measured 3 months following discontinuation of anticoagulation therapy [24, 25]. The association of d-dimer and VTE recurrence is significant among both non-carriers (HR 2.43; 95 % CI 1.18–4.61) and carriers of thrombophilia (HR 8.34; 95 % CI 2.72–17.43) [26]. In a systematic review of seven studies involving 1888 patients with a first unprovoked episode of VTE, those with an elevated d-dimer measured following withdrawal of anticoagulation therapy had an annual rate of VTE recurrence of 8.9 % compared to 3.5 % among patients with a normal d-dimer [27]. In a separate patient level meta-analysis of the same population, the annual rates of VTE recurrence were 8.8 and 3.7 % for patients with elevated and normal d-dimer, respectively [28].

## d-Dimer and mortality

An elevated d-dimer is associated with short and long term increased mortality [12, 29]. In a meta-analysis of five studies (n = 2,885) of patients with pulmonary embolism, an elevated d-dimer was associated with increased all cause 30 day mortality (OR 2.76; 95 % CI 1.83–4.14). Furthermore, in a 4 study meta-analysis (n = 1,254) an elevated d-dimer was associated with a higher 3-month mortality (OR 4.29; 95 % CI 1.70–10.79) [29].

The prognostic significance of d-dimer among stroke patients is mixed. Although one study did not find an association between d-dimer and mortality [30], others reported that d-dimer is elevated among stroke patients who suffer a subsequent vascular death (*p* = 0.0201) [31]. Furthermore, an elevated d-dimer within the first weeks of ischemic stroke is associated with poorer survival independent of age, stroke type, or severity (HR 6.43; 95 % CI 2.83–14.62; *p* < 0.0001) [32].

Among cancer patients, d-dimer may be elevated not only in the presence of documented VTE but also in the absence of any detectable thrombus [33]. Elevated d-dimer is associated with an increased risk of mortality in a variety of cancers independent of age, sex, documented VTE, and types of malignancy [17, 33, 34]. In a meta-analysis of 13 studies of lung cancer patients in particular, elevation of d-dimer was associated with increased mortality (HR 2.06; 95 % CI 1.64–2.58; *p* = 0.0001) [35].

Like cancer, d-dimer is associated with higher mortality among patients with cardiovascular disease. In a study of 174 patients with heart failure, a d-dimer >1,435 ng/mL was associated with increased cardiovascular mortality (HR 3.25; 95 % CI 1.647–6.414; *p* = 0.001) [36]. Moreover, in the AtheroGene Study of 1,057 patients with coronary artery disease prospectively followed for a median of 6.6 years, an elevated d-dimer was independently associated with high cardiovascular mortality [37]. Among patients with aortic dissection, an elevated d-dimer is associated with increased hospital mortality [38, 39].

In the setting of community acquired pneumonia and sepsis, elevation in d-dimer is independently associated with increased mortality [40–42]. Finally, even in the absence of clinically overt disease, among 17,359 subjects free of cardiovascular disease and cancer from the MOLI-SANI prospective cohort study, an elevated d-dimer was associated with increased mortality over the subsequent 4 years [43].

## Limitations

Although multiple studies have demonstrated an association between elevated d-dimer and prognosis, no single cut-point has been identified which consistently optimizes the prognostic value of the biomarker. The cut-point evaluated in multiple studies is that which exceeds the conventional d-dimer cut-off value (500 ng/mL). However, the threshold for d-dimer exceeded 5,500 ng/mL in one study [44]. d-Dimer concentrations were measured only once in the majority of the studies.

Comparing clinical outcomes between studies which utilized different d-dimer assays and cut-points is challenging. d-dimer assays are not standardized, and some assays express d-dimer in fibrin equivalent units (FEU), while others express it in d-dimer units (one d-dimer unit is approximately two FEU) [33]. Moreover, d-dimer is also influenced by age [45] and concomitant medical conditions, such as atrial fibrillation, heart failure, peripheral artery disease and renal failure [36, 46–49]. The elevation in d-dimer in these patient groups is a limitation for both the diagnostic and prognostic role of d-dimer, particularly among the elderly and patients with renal failure among whom prolonged VTE prophylaxis can be problematic due to excess inadvertent bleeding [50, 51].

Finally, the outcomes of the studies regarding the occurrence of VTE events were not consistent across different studies as some included asymptomatic DVT while others did not. In addition, not all studies ruled out the presence of asymptomatic thrombus at the time of d-dimer measurement, and the possibility that the subsequent VTE episode was due to an existing clot cannot be excluded.

## Conclusion

d-Dimer elevation is associated with the risk of first VTE occurrence, VTE recurrence, and mortality. An elevated d-dimer is an independent correlate of increased mortality and subsequent VTE across a broad variety of disease states. Therefore, medically ill subjects in whom the d-dimer is elevated constitute a high risk subgroup in which the prospective evaluation of the efficacy and safety of antithrombotic therapy is warranted.

## Abbreviations

VTE: Venous thromboembolism
PE: Pulmonary embolism
DVT: Deep vein thrombosis
